# Community-associated Methicillin-resistant *Staphylococcus aureus*, Canada

**DOI:** 10.3201/eid1106.041146

**Published:** 2005-06

**Authors:** Michael R. Mulvey, Laura MacDougall, Brenda Cholin, Greg Horsman, Melanie Fidyk, Shirley Woods, the Saskatchewan CA-MRSA Study Group

**Affiliations:** * National Microbiology Laboratory, Winnipeg, Manitoba, Canada;; † British Columbia Centre for Disease Control, Vancouver, British Columbia, Canada;; ‡ Health Canada, Ottawa, Ontario, Canada;; § Prairie North Health Region, North Battleford, Saskatchewan, Canada;; ¶ Saskatchewan Health Provincial Laboratory, Regina, Saskatchewan, Canada;; # Kelsey Trail Health Region, Nipawin, Saskatchewan, Canada;; ** Northern Intertribal Health Authority, Prince Albert, Saskatchewan, Canada

**Keywords:** antimicrobial resistance, MRSA, community-associated

## Abstract

A total of 184 methicillin-resistant *Staphylococcus aureus* (MRSA) strains were collected from patients who sought treatment primarily for skin and soft tissue infections from January 1, 1999, to March 31, 2002, in east-central Saskatchewan, Canada. Molecular subtyping analysis using pulsed-field gel electrophoresis showed 2 major clusters. Cluster A (n = 55) was composed of a multidrug-resistant MRSA strain associated with a long-term care facility and was similar to the previously reported nosocomial Canadian epidemic strain labeled CMRSA-2. Cluster B (n = 125) was associated with cases identified at community health centers and was indistinguishable from a community-associated (CA)-MRSA strain identified previously in the United States (USA400). Cluster B remained susceptible to a number of classes of antimicrobial agents and harbored the *lukF*-*PV* and *lukS*-*PV* toxin genes. Over 50% of both clonal groups displayed high-level resistance to mupirocin. This is the first report of the USA400 strain harboring the *lukF*-*PV* and *lukS*-*PV* toxin genes in Canada.

The first report of a highly virulent community-acquired methicillin-resistant *Staphylococcus aureus* (CA-MRSA) strain occurred in 1993 in Australia (1), and since that time CA-MRSA has been reported in many countries. CA-MRSA strains are different genetically and epidemiologically from strains commonly associated with nosocomial infections. Common nosocomial risk factors generally do not apply to CA-MRSA, although previous antimicrobial drug use has been identified as a potential risk factor for CA-MRSA (2–4). In addition, reports have documented CA-MRSA as having caused serious, and sometimes fatal, disease, especially in otherwise healthy children (5–7). Most CA-MRSA strains remain susceptible to a number of classes of antimicrobial agents such as aminoglycosides, tetracyclines, and fluoroquinolones. Many reports of CA-MRSA have described strains harboring the Panton-Valentine leukocidin determinant, a virulence factor for primary skin infection and pneumonia (8,9).

Over the past decade, MRSA has been observed sporadically as a community-acquired pathogen in Canada (10,11). On the Canadian prairies a disproportionate number of aboriginal persons admitted to acute care facilities are infected or colonized with MRSA, compared to persons of nonaboriginal origin (10,12).

This report describes the emergence of 2 different strains of MRSA in east-central Saskatchewan, Canada. The first was associated with a long-term care facility and the second was a clone of MRSA harboring the *lukF*-*PV* and *lukS*-*PV* toxin genes and generated an indistinguishable fingerprint from the previously described USA400 strain. This is the first report describing the emergence in Canada of the strain of USA400 that contains *lukF*-*PV* and *lukS*-*PV*.

## Materials and Methods

### Setting

The investigation focused on an area in east-central Saskatchewan. This region consisted of a city of ≈7,127 persons that was serviced by a hospital with a central laboratory in which the MRSA strains were identified. This locale also contained a number of community health centers and a long-term care facility housing ≈100 persons. The region also included a number of smaller communities that consisted of small rural First Nations and Metis communities (aboriginal populations) and small towns.

### Case Definitions

Surveillance for MRSA was laboratory-based and involved nonrepeat MRSA cases. Case-patients were residents of east-central Saskatchewan with laboratory-confirmed MRSA infections identified from January 1, 1999, to March 31, 2002, by the local hospital or provincial laboratory. All MRSA isolates identified by the hospital laboratory were subsequently confirmed at the provincial laboratory by using standard protocols. Residential status was determined by the location of the patient's treatment facility because case-patient address information was unavailable. General patient demographic information was collected (date of birth, sex, date of sample collection, and invasiveness) for all case-patients. Invasive infections were defined according to the guidelines from the Centers for Disease Control and Prevention Active Bacterial Core Surveillance Program and included obtaining isolates from a normally sterile site, such as blood, cerebrospinal fluid, pleural fluid, peritoneal fluid, pericardial fluid, surgical aspirate, bone, joint fluid, or internal body site (www.cdc.gov/ncidod/dbmd/abcs/meth-case.htm). Antimicrobial susceptibility testing by microbroth dilution was performed according to NCCLS recommendations (13). Breakpoints used for mupirocin were as follows: susceptible, MIC <4 mg/L; low-level resistance, MIC ≥4 and <256 mg/L; high-level resistance, ≥256 mg/L (14).

### Molecular Characterization of MRSA Strains

*mecA* and *nuc* genes from MRSA isolates were coamplified with a multiplex real-time polymerase chain reaction (PCR) assay. Nucleic acid was isolated from 4–5 colony picks by boiling in a 2% (wt/vol) homogeneous suspension of Chelex 100 resin (Bio-Rad Laboratories Ltd, Mississauga, Ontario, Canada). Primer and probe sequences with their reaction concentrations are shown in Table 1. Master mix was composed of the Applied Biosystems TaqMan PCR Core kit (Applied Biosystems, Foster City, CA, USA) that uses reaction concentrations of: 1× PCR buffer A, 4.0 mmol/L MgCl_2_, 200 μmol/L dATP, 200 μmol/L dCTP, 200 μmol/L dGTP, 400 μmol/L dUTP, 1.25 U AmpliTaq Gold, and 0.5 U uracil-DNA-N-glycosylase (UNG) for carryover prevention. Thermal cycling and data collection were performed on an ABI Prism 7700 Sequence Detector using the following conditions: 2 min at 50°C, 10 min at 95°C, followed by 55 cycles of 95°C for 15 s and 60°C for 1 min. Amplification was confirmed for each target by the generation of a sigmoid amplification plot.

**Table 1:** DNA oligonucleotides used in this study

Primer/Probe	Oligonucleotide sequence	Final concentration (μmol)
*mec*A forward	5´ GGC AAT ATT ACC GCA CCT CA 3´	0.30
*mec*A reverse	5´ GTC TGC CAC TTT CTC CTT GT 3´	0.30
*mec*A probe	5´ FAM - AGA TCT TAT GCA AAC TTA ATT GGC AAA TCC - TAMRA 3´	0.10
*nuc* forward	5´ CAA AGC ATC AAA AAG GTG TAG AGA 3´	0.05
*nuc* reverse	5´ TTC AAT TTT CTT TGC ATT TTC TAC CA 3´	0.05
*nuc* probe	5´ VIC - TTT TCG TAA ATG CAC TTG CTT CAG GAC CA - TAMRA 3´	0.05

*lukF*-*PV* and *lukS*-*PV* detection was carried out by using PCR with primers and protocols previously described (15). PCR was used to detect the exfoliative toxin genes *eta* and *etb* as previously described (16).

Isolates were subtyped by using pulsed-field gel electrophoresis (PFGE) after digestion with *Sma*I following the Canadian Standardized Protocol as described previously (17). PFGE-generated DNA fingerprints were digitized and analyzed with BioNumerics Ver. 3.5 (Applied Maths, Sint-Martens-Latem, Belgium) by using a position tolerance of 1.0 and an optimization of 1.0. Relatedness was determined following established criteria with major strain clusters (designated by a letter) grouped with banding patterns of <7 band changes (18). Fingerprints were compared to those in the national MRSA fingerprint database, which comprised of >600 unique MRSA fingerprint types. Isolates with specific DNA profiles were grouped into 1 of 6 Canadian epidemic strains of MRSA (CMRSA-1, CMRSA-2, CMRSA-3, etc.) as previously described (19,20). Multilocus sequence typing (MLST) was conducted on a representative isolate from each of the unique PFGE types as previously described (21). Staphylococcal chromosome cassette (SCC) *mec* typing was conducted as previously described (22).

## Results

### Epidemiologic Analysis of MRSA

**Figure 1:**
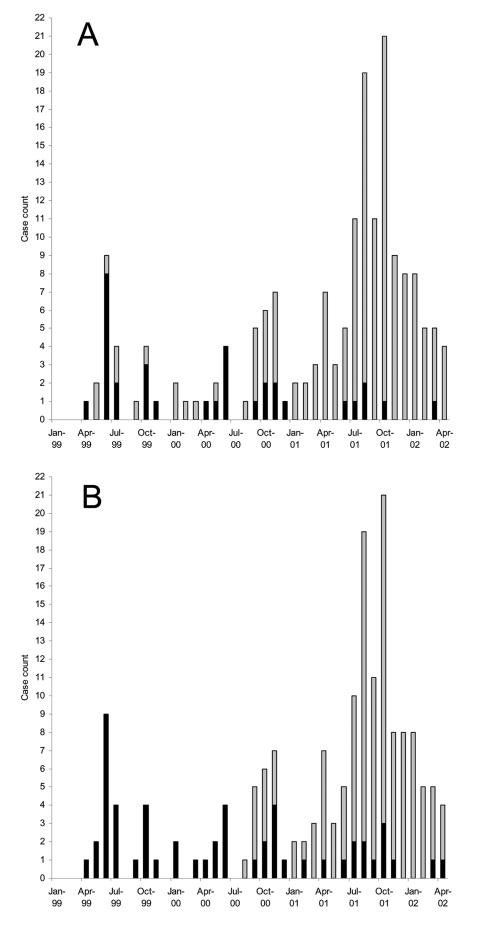
Epidemiologic curve showing the emergence of methicillin-resistant *Staphylococcus aureus* (MRSA) in central-eastern Saskatchewan. A) Number of nonrepeat cases over the length of study; solid bars, cases identified in a long-term care facility; gray bars, cases identified in community health centers. B) Same data as (A), with solid bars representing isolates of clone A and gray bars showing isolates of clone B.

Before 1999, MRSA was rarely isolated in east-central Saskatchewan. It was identified only 2 other times in persons from this region since 1996. However, in April 1999, a resident of a long-term care facility in the region under study tested positive for MRSA. This patient had recently been hospitalized in Saskatoon, Saskatchewan, and tested positive for MRSA shortly after being transferred back to the long-term care facility. Following this case, an additional 183 nonrepeat MRSA were isolated from infected persons from this region between January 1999 and April 2002. The annual rates of MRSA-related infections in the region under study were 3.1/1,000 persons in 1999, 4.8/1,000 persons in 2000, and 14.6/1,000 persons in 2001. In 1 specific community within the study area with a population of approximately 1,400 persons, MRSA rates were 0/1,000 in 1999, 7.1/1,000 in 2000, and 46/1,000 in 2001. Only a single isolate was considered invasive, having causes a blood infection. Approximately 18% (n = 33) of the cases were identified from a single long-term care facility and made up of >50% of cases identified between April 1999 and June 2000 (Figure 1A). After June 2000, a larger cluster of cases emerged, which peaked in October 2001 and were primarily identified from patients presenting at local health clinics or nursing stations from surrounding communities (Figure 1A).

### Molecular Characterization of MRSA

**Figure 2:**
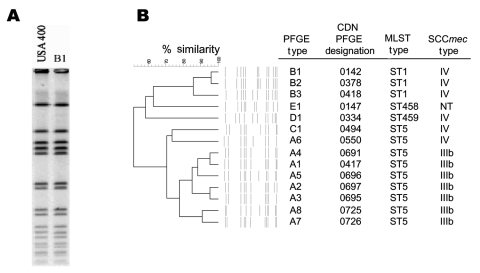
A) Pulsed-field gel electrophoresis (PFGE) fingerprint of USA400 and PFGE pattern B1. B) Dendrogram showing relationship of the unique fingerprints, along with the PFGE type designation (11) and other molecular characteristics of each subtype. CDN, Canadian Diseases Network; MLST, multilocus sequence typing; SCC, staphylococcal chromosome cassette.

All of the 184 strains were PCR positive for the *mec*A and *nuc* genes, respectively. DNA fingerprinting of all strains using PFGE resulted in the identification of 5 major fingerprint patterns labeled pattern A to E (Figure 2). Clonal group A (n = 55) comprised 8 subtypes with subtype A1 representing 86% (n = 47, Canadian Diseases Network (CDN) type 417) of the total, with other A subtypes as follows: A2 (n = 1, CDN type 697); A3 (n = 1, CDN type 695); A4 (n = 2, CDN type 691); A5 (n = 1, CDN type 696); A6 (n = 1, CDN type 550); A7 (n = 1, CDN type 726); A8 (n = 1, CDN type 725). Group B was the most predominant molecular subtype with 68% (n = 126) of the strains in this cluster. This group was comprised of 3 PFGE subtypes labeled B1 (n = 123, CDN type 142), B2 (n = 1, CDN type 378), and B3 (n = 2, CDN type 418). Three additional unique PFGE fingerprint types were labeled C1 (n = 1, CDN type 494), D1 (n = 1, CDN type 334), and E1 (n = 1, CDN type 147).

### Characterization of Clonal Group A Isolates

**Figure 3:**
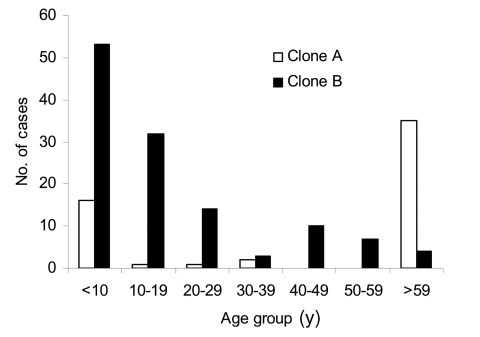
Age distribution of patients with cases caused by each clonal type A (white bars) or type B (black bars).

Clonal group A isolates were first identified on April 12, 1999, from a patient in a long-term care facility who had been transferred from a hospital in Saskatoon, Saskatchewan. A rapid increase in cases was related to this clonal group from persons from the long-term care facility (n = 33, 60%) as well as community health centers (n = 22, 40%) during the next few months (Figure 1B). Cases related to this group peaked June 1999; however, isolates continued to be identified over the course of this study from both patients in long-term care facilities and community health centers (Figure 1). Clonal group A strains were found to cause a large number of noninvasive infections. Only a single isolate was identified as causing an invasive blood infection. A slightly higher number of female patients 58% (n = 32) were identified with clonal group A–related infections. Approximately 64% (n = 35) of these strains were identified from persons >59 years of age, although 29% (n = 16) of cases were identified in persons <10 years of age (Figure 3). Of the 22 cases reported from the community health centers, 73% (n = 16) were <18 years of age. Comparison of the representative PFGE clonal group A1 fingerprint pattern to the Canadian National Fingerprint Database showed that these strains were similar to the previously identified epidemic MRSA-labeled CMRSA-2 (19,20). A representative strain from each of the unique fingerprint patterns from the A clonal group was typed by using MLST, and all were found to be of the sequence type ST5. All of the unique A subtypes as well as a 25% (n = 12) sample of strains that were of the A1 subtype did not harbor the *lukF*-*PV*, *lukS*-*PV*, *eta*, and *etb* toxin genes. SCC*mec* typing of 4 A1 subtypes and subtypes A2, A3, A4, A5 showed all contained SCC*mec* IIIb. Most clonal group A isolates were resistant to ciprofloxacin, erythromycin, gentamicin, tetracycline, mupirocin, and fusidic acid (Table 2).

**Table 2:** Antimicrobial susceptibilities of the different clonal groups identified using pulsed-field gel electrophoresis

Antimicrobial agent	Clonal group A (n = 55)				Clonal group B (n = 126)			
	MIC_50_ (mg/L)	MIC_90_ (mg/L)	% resistant	Range	MIC_50_ (mg/L)	MIC_90_ (mg/L)	% resistant	Range
Oxacillin	16	32	100	4–64	16	32	100	4–64
Cefazolin	32	32	75	4–32	16	32	31	2–32
Ciprofloxacin	32	≥32	86	0.25–≥32	0.25	0.25	0	0.25–0.5
Clindamycin	<0.25	≥8	11	<0.25–≥8	<0.25	<0.25	1.6	<0.25–≥8
Erythromycin	>8	≥8	87	<0.25–≥8	<0.25	≥8	40	<0.25–≥8
Gentamicin	>16	≥16	64	<0.5–≥16	<0.5	<0.5	0	<0.5
Rifampin	<0.25	<0.25	3.6	<0.25–≥4	<0.25	<0.25	0	<0.25
Tetracycline	16	≥16	55	<2–≥16	<2	<2	0	<2
Trimethoprim/sulfamethoxazole	<0.25	0.5	5.5	<0.25	<0.25	<0.25	0	<0.25
Vancomycin	1.0	1.0	0	1.0	1.0	1.0	0	0.5-1.0
Linezolid	4.0	4.0	0	4.0	2.0	4.0	0	1.0-4.0
Mupirocin	≥128	≥128	58	≥128	≥128	≥128	55.6	<0.25–≥128
Fusidic acid	≥8	≥8	100	≥8.0	0.12	0.25	0	0.12–0.25

### Characterization of Clonal Group B Isolates

The first reported case of infection due to clonal group B occurred 16 months after the description of first reported MRSA infection on August 31, 2000, and the cases continued to increase with a peak of occurring in October 2001 (Figure 1B). Clonal group B correlated with a large number of noninvasive infections reported from community health centers, and only 1 report was made of a long-term care patient with MRSA type 0142 infection. The distribution by sex was similar; 49% of patients were female (n = 62) and 51% were male (n = 64). More than 67% (n = 85) of these strains were identified from persons <20 years of age, although ≈17% (n = 21) of cases were identified in persons >40 years (Figure 3).

The PFGE B1 fingerprint pattern (CDN type 142) was indistinguishable from the USA CA-MRSA strain labeled USA400 (Figure 2) (21). MLST was conducted on 1 representative strain from each clonal group B subtype, and all were the same sequence type (ST1). In addition, these MRSA strains were shown to contain SCC*mec* type IV. A selection of ≈25% of the PFGE type 142 strains (n = 33), including all of the unique fingerprint patterns B2 and B3, was shown by PCR to harbor the *lukF*-*PV* and *lukS*-*PV* genes, and none of the strains tested harbored the *eta* and *etb* toxin genes. Clonal group B strains were susceptible to most antimicrobial agents tested; however, 40% and 55% of the isolates displayed resistance to erythromycin and high-level resistance to mupirocin, respectively (Table 2).

### Characterization of Other Molecular Subtypes

Three other unique PFGE subtype groups were identified; these were composed of single isolates labeled C1, D1, and E1, respectively (Figure 2). None of these isolates carried the *lukF*-*PV* and *lukS*-*PV* toxin genes or the exfoliative toxins. Similar to the PFGE B clonal group, these strains remained susceptible to most non–β-lactam drugs tested (data not shown).

## Discussion

A dramatic increase of MRSA in the east-central region of Saskatchewan has been documented in this study. Molecular typing analysis has shown that 2 major unrelated strain clusters were in circulation in the communities under study. The emergence of clonal group A was linked to a patient in a long-term care facility who had recently been transferred from a hospital in Saskatoon, and this strain began to spread within the facility. Comparisons to the Canadian National Fingerprint Database showed the A1 pattern is related to the CMRSA-2 epidemic strain cluster observed in Canadian tertiary care hospitals (19,20). Forty percent of infections caused by the A clonal group were identified from community health centers, which suggests that this strain may have spread from the long-term care facility into the community. In fact, CMRSA-2 has been previously shown to be more likely to be associated with community isolations than the other epidemic CMRSA clusters (19). Unlike previously described CA-MRSA strains, this strain does not harbor the PVL or exfoliative toxin genes, and it displays a multidrug resistance phenotype. However, analysis of the community health center cases showed that most (73%) were from persons <18 years of age. Current studies that use comparative genomics are under way in our laboratory to determine whether any genetic differences exist between community, nosocomial, and sporadic MRSA that may explain the association with community isolation (23).

The more predominant clone (cluster B) in this study was, with 1 exception, identified from cases reported from community health centers. Comparison of the B1 fingerprint pattern showed that it was indistinguishable to the USA 400 strain, which is responsible for CA-MRSA infections in the midwestern United States (24,25). Although the source of the USA 400 strain into this community cannot be determined, a community-based cluster of MRSA was reported in a rural community in southwestern Manitoba in 1997, and this strain had been identified previously from persons in northwestern Manitoba since 1995 (26). We have demonstrated that this strain is also indistinguishable from the USA400 fingerprint pattern (data not shown). This strain may have spread from northwestern Manitoba to the community in this current study because they are geographically close (J. Wylie, pers. comm.).

When all of the typing information is compared, some observations warrant mention. The single isolate with the molecular subtype designated A6 contains SCC*mec* IV, which is different from the other A-subtypes that contain SCC*mec* IIIb. Notably, all A-subtypes are clustered into the MLST group ST5. Although the SCC*mec* IV region is smaller than that of the SCC*me*c IIIb, it could not have arisen from a simple deletion event because sequence analysis of these 2 cassettes has shown l significant difference that cannot be explained by a simple deletion event (27). One possible explanation is that a recombinational event occurred, which led to the replacement of SCC*mec* IIIb with the SCC*mec* IV. Instability in these regions has been reported, although the events are likely rare (28,29). Since clone B harboring the SCC*mec* IV is also circulating in this community, a recombination event may have occurred. Furthermore, the MRSA strains with macrorestriction patterns C1 and D1 also contained the SCCmec IV region, which suggests that horizontal transfer of this cassette to additional *S. aureus* strains may have occurred. Alternatively, the strain with the A6 pattern may have emerged independently from the other strains within the A cluster.

This study documents the finding of high-level mupirocin resistance in >50% of all study strains (clones A and B). Mupirocin can be used to treat superficial skin infections and has been used to decolonize patients. High-level mupirocin resistance in MRSA was first described in 1996 among patients in a burn unit in Kuwait (30). Mupirocin resistance is mediated by the *mupA* gene, which is generally plasmid encoded, although recently the gene has been identified on the chromosome of *S*. *aureus* (30,31). Since high-level resistance has been documented on plasmids that vary in size and restriction patterns (1,32), examining the plasmid restriction fragment length polymorphisms may be useful in monitoring monitor plasmid dissemination between and within the clonal types. In a similar manner, we previously described extended-spectrum β-lactamase–containing plasmids in gram-negative organisms (33). Information from this exercise could determine if horizontal transfer of the gene coding for mupirocin resistance occurred between the two clonal groups A and B.

We retrospectively reviewed a sample of charts from patients with skin and soft tissue infections in 2002 in the region of highest CA-MRSA frequency and found that 18% of these infections resulted in a prescription for mupirocin (unpub. data). The use of mupirocin in these communities may be driving resistance. A recent study of patients from a Tennessee medical center documented that decreased usage of mupirocin reduces this form of resistance in MRSA (33). We are currently developing educational programs to decrease the use of this drug, which we hope will decrease mupirocin resistance in this region.

The 2 clonal groups of MRSA described in this study continue to circulate in this area of Saskatchewan (data not shown). We are currently undertaking a case-control study to identify risk factors associated with infections caused by these 2 strains in the community. We call for future studies to include mupirocin in their antimicrobial resistance panels because resistance to this agent may affect treatment outcomes.
